# The Genetic Basis of Pollinator Adaptation in a Sexually Deceptive Orchid

**DOI:** 10.1371/journal.pgen.1002889

**Published:** 2012-08-16

**Authors:** Shuqing Xu, Philipp M. Schlüter, Ueli Grossniklaus, Florian P. Schiestl

**Affiliations:** 1 Institute of Systematic Botany and Zürich-Basel Plant Science Center, University of Zürich, Zürich, Switzerland; 2 Institute of Integrative Biology and Zürich-Basel Plant Science Center, ETH Zürich, Zürich, Switzerland; 3 Institute of Plant Biology and Zürich-Basel Plant Science Center, University of Zürich, Zürich, Switzerland; Iowa State University, United States of America

## Abstract

In plants, pollinator adaptation is considered to be a major driving force for floral diversification and speciation. However, the genetic basis of pollinator adaptation is poorly understood. The orchid genus *Ophrys* mimics its pollinators' mating signals and is pollinated by male insects during mating attempts. In many species of this genus, chemical mimicry of the pollinators' pheromones, especially of alkenes with different double-bond positions, plays a key role for specific pollinator attraction. Thus, different alkenes produced in different species are probably a consequence of pollinator adaptation. In this study, we identify genes that are likely involved in alkene biosynthesis, encoding stearoyl-acyl carrier protein (ACP) desaturases (SAD), in three closely related *Ophrys* species, *O. garganica*, *O. sphegodes*, and *O. exaltata*. Combining floral odor and gene expression analyses, two *SAD* homologs *(SAD1/2)* showed significant association with the production of (*Z*)-9- and (*Z*)-12-alkenes that were abundant in *O. garganica* and *O. sphegodes*, supporting previous biochemical data. In contrast, two other newly identified homologs *(SAD5/6)* were significantly associated with (*Z*)-7-alkenes that were highly abundant only in *O. exaltata*. Both molecular evolutionary analyses and pollinator preference tests suggest that the alkenes associated with *SAD1/2* and *SAD5/6* are under pollinator-mediated divergent selection among species. The expression patterns of these genes in F_1_ hybrids indicate that species-specific expression differences in *SAD1/2* are likely due to *cis*-regulation, while changes in *SAD5/6* are likely due to *trans*-regulation. Taken together, we report a genetic mechanism for pollinator-mediated divergent selection that drives adaptive changes in floral alkene biosynthesis involved in reproductive isolation among *Ophrys* species.

## Author Summary

In plants, the extraordinary floral diversity has been suggested to be a consequence of divergent adaptation. However, the genetic basis of this process is poorly understood. In this study, we take advantage of the high specificity of plant-pollinator interactions in the sexually deceptive orchid genus *Ophrys*. We leverage the available, ample evidence showing that floral odors, especially alkenes, are the key factor for specific pollinator attraction in certain species of these orchids. Further, we investigate the genetic basis of pollinator adaptation. By applying an inter-disciplinary approach, including chemical ecology, gene expression analysis, population genetics, and pollinator-behavioral tests, we show that genetic changes in different copies of a biosynthetic gene are associated with the production of different floral scents and with pollinator adaptation in these orchid species. Moreover, we found that both *cis*- and *trans*-regulatory factors are likely involved in controlling gene expression of these biosynthetic gene copies. These findings support the hypothesis that adaptation is mediated by very few genetic changes with large phenotypic effects, rather than requiring a large number of co-adapted genes.

## Introduction

Understanding the genetic basis of adaptation is of great interest to evolutionary biologists. For over a century, it has been debated whether adaptations are likely caused by a large number of mutations of small phenotypic effect or by very few genetic changes of large effect [1]–[3]. To address this question, it is necessary to identify the genetic basis of adaptive traits and their ecological significance in any given study system [4].

Pollinator-mediated selection on floral traits has been considered to be a major driving force of floral diversification and speciation in plants [4]–[9]. Closely related species featuring distinct floral traits, such as floral color, odor, or spur lengths, are widely thought to be a consequence of pollinator adaptation [4], [6], [8]–[11]. Furthermore, pollinator adaptation often conveys reproductive isolation [12], [13], and thus may directly contribute to the origin of novel species. Therefore, floral traits associated with pollinator adaptation are of special interest for the understanding plant speciation and evolution.

*Ophrys* orchids mimic their pollinators' mating signals and are pollinated by male insects during mating attempts with the flower. This pollination by so-called sexual deception is very specific, and each orchid species only attracts one or very few insect species [14], [15]. Specific pollinator attraction has been reported to be the main reproductive barrier in *Ophrys* [15]–[17]. The key to specific pollinator attraction is the chemical mimicry of the insect female's sex pheromone [11], [18]–[22], usually a blend of cuticular hydrocarbons, namely alkanes and alkenes. Among these, alkenes with different double-bond position are particularly important for the specificity of pollinator attraction [11], [20], [22]. Thus, genes specifying alkene double-bond positions may be directly associated with pollinator adaptation.

In plants, alkene double-bonds are likely determined by desaturases such as stearoyl-ACP (acyl carrier protein) desaturases (SADs) [23]–[25]. At the onset of alkene biosynthesis, desaturases can insert a *cis*-double-bond into a saturated fatty acid (FA) intermediate, such as 16:0-ACP (C:D denotes a fatty acyl group of C carbons length with D double-bonds), to produce an unsaturated FA such as 16:1ω-7-ACP or 16:1ω-9-ACP (double-bond at position ω-7 or ω-9, counting from the aliphatic end). Unsaturated FA intermediates are elongated from the ACP end [26], leading to the production of (*Z*)-7- or 9-alkenes from ω-7 or ω-9 FA intermediates, respectively [23], [24] (Since all alkenes in this study are presumed to be in the *cis* (*Z*) configuration, only double-bond position will be indicated for alkenes in the following text). Therefore, changes in SAD enzyme activity or *SAD* gene expression may result in alkene double-bond position differences among species [25].

In this study, we focused on three closely related sympatric species of the genus *Ophrys: O. garganica*, *O. sphegodes*, and *O. exaltata*, among which the phylogenetic relationship could not yet be resolved by neutral sequence markers [27]. These three co-flowering species largely overlap in their geographical distributions and are similar in floral morphology [28], [29], however, they are reproductively isolated from each other due to the attraction of different pollinators [17]. Although the color of floral petals also varies among species, the key factor for differential pollinator attraction is floral scent [30]. Flowers of both *O. garganica* and *O. sphegodes* produce a high proportion of 9- and 12-alkenes (with differences in carbon chain length), whereas *O. exaltata* produces high amounts of 7-alkenes. These 7-alkenes have previously been shown to be important for attraction of the bee, *Colletes cunicularius*, the pollinator of *O. exaltata* [19], [31]. We have previously shown that in *O. sphegodes*, 9- and 12-alkene production is associated with the *SAD2* gene, which encodes a functional desaturase [24]. However, the genetic basis of 7-alkene biosynthesis in *O. exaltata* and the driving forces for evolutionary divergence in alkene production among species are unknown. Here we investigate gene expression and evolutionary relationships among *SAD* gene family members in different natural orchid populations and species. Moreover, we discuss the biosynthetic origin of 7-alkenes as well as the role of pollinator-mediated selection in changing alkene composition among species. Specifically, we address the following questions: (a) Which desaturase is responsible for 7-alkene biosynthesis? (b) What is the role of pollinator-mediated selection in the evolution of alkene production? (c) How is the biosynthesis of different alkenes by different *Ophrys* species regulated genetically?

## Results

### *Stearoyl-ACP-desaturase* homologs in *Ophrys*

**Figure 1:**
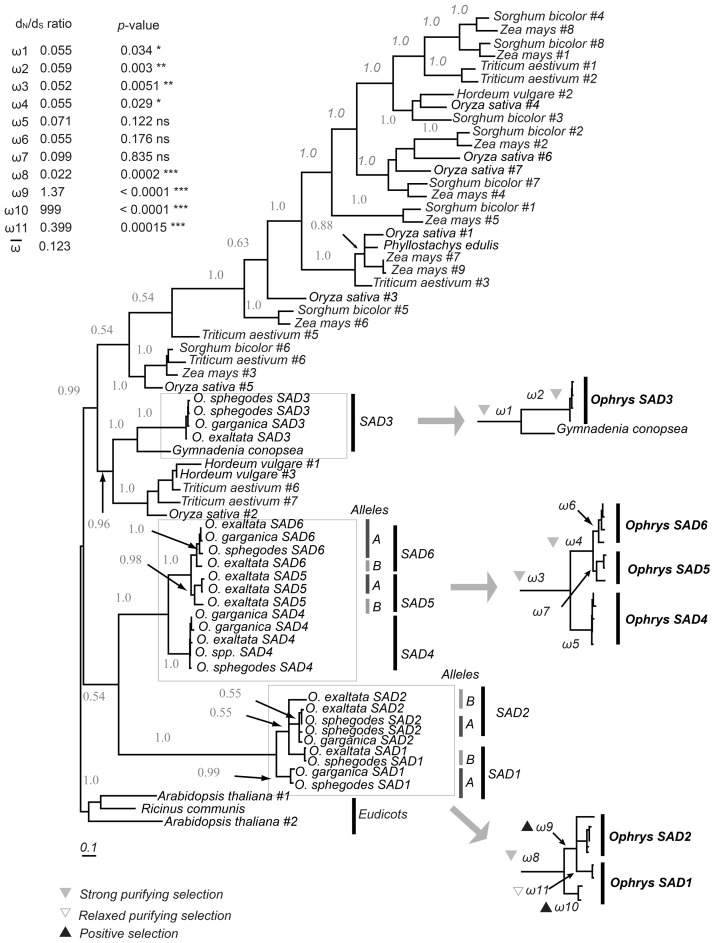
Bayesian inference phylogenetic tree of monocot *SAD* homologs and eudicot outgroup. Numbers indicate posterior probabilities (where >0.5) above branches. *Ophrys SAD* homologs and their assignment to different allele groups are highlighted and ω = *d*_N_/*d*_S_ ratios for branches of interest indicated. A black upward triangle indicates significant positive selection; a gray downward triangle indicates purifying selection, and a non-filled triangle indicates relaxed purifying selection. The inset lists the ω-values, associated *p*-value, and significance (*, *p*<0.05; **, *p*<0.01; ***, *p*<0.001).

We identified six *SAD* homologs, which we named *SAD1–SAD6*. Three of these (*SAD1–3*) have been described previously [24]; *SAD4–6* were identified by homology from high-throughput transcriptome sequencing of *O. sphegodes* flowers. Phylogenetic analysis of plant *SAD* genes indicated that all six *Ophrys* homologs evolved via gene duplication, forming three distinct lineages, *SAD1/2*, *SAD3*, and *SAD4/5/6*. The split of *SAD1/2* from *SAD4/5/6* was more recent than the split of these groups from *SAD3* (Figure 1).

To test for the signature of selection, a codon-based analysis comparing the rates of synonymous and non-synonymous mutations was performed. It revealed a significant signal indicative of positive (ω_9_ and ω_10_) and relaxed purifying selection (ω_11_, all *p*<0.001, Figure 1) after the split of *SAD1* and *SAD2*, concordant with previous findings [24]. However, no indication of positive selection was found for any other *SAD* locus or clade. Purifying selection significantly stronger than the background rate was found on the *SAD3* branch, as well as prior to the split of *SAD1/2*, and prior to the split of *SAD4/5/6*.

### Expression of certain *SAD* homologs is correlated with alkene production during development

**Figure 2:**
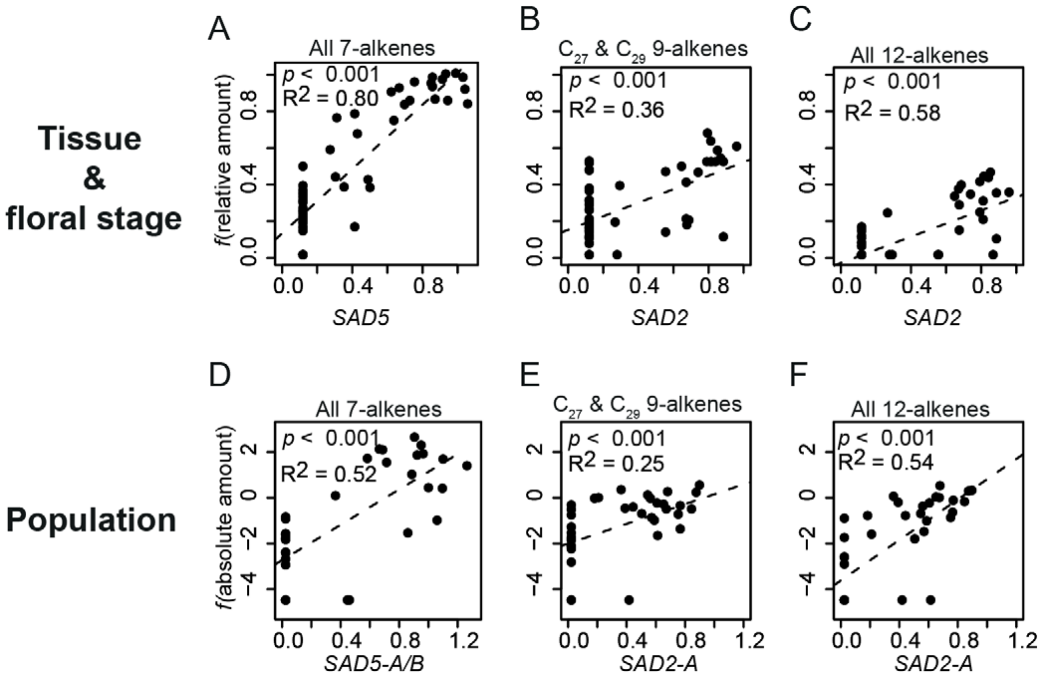
Regression of normalized desaturase gene expression with alkenes. (*A–C*) Relative amount of alkenes was used after *f(x) = arcsin x^0.5^* transformation for tissues and floral stages of *O. exaltata* (*A*) and *O. sphegodes* (*B,C*). (*D–F*) Absolute amount of alkenes (in µg) was used after *f(x) = ln (x+0.01)* transformation for population data from three species. For both datasets, normalized *SAD* expression was used after *f(x) = x^0.5^* transformation. Adjusted R^2^ is indicated in each graph. (*A,D*) Correlation of all (C_21_-C_29_) 7-alkenes with *SAD5*; (*B,E*) Correlation of C_27_+C_29_ 9-alkenes with *SAD2* expression; (*C,F*), Correlation of all (C_25_-C_29_) 12-alkenes with *SAD2* expression.

Among the six *SAD* homologs investigated for tissue- and floral developmental stage-specific expression, five (*SAD1–5*) were found to be expressed in the 11 tested greenhouse-grown individuals. Four homologs (all except *SAD3*) showed flower-specific expression (Figure S1). The expression levels of *SAD2* and *SAD5* were significantly associated with the presence of alkenes: *SAD2* expression was significantly (*p*<0.001) associated with both 12- and 9-alkenes, whereas *SAD5* expression was significantly (*p*<0.001) associated with 7-alkenes in *O. exaltata* across different tissues and floral developmental stages (Figure 2).

### Allelic variation of *SAD* homologs among species

All *SAD* homologs except *SAD3* and *SAD4* showed species-specific patterns of allelic variation among the three studied orchid species (Figure S2). Two allele groups were found for each *SAD1* (*SAD1-A/B*), *SAD5* (*SAD5-A/B*) and *SAD6* (*SAD6-A/B*), whereas four allele groups were found for *SAD2* (*SAD2-A/B/C/D*). Among these *SAD* allele groups, biochemical activity assays suggest that *SAD1-B* and *SAD2-C* alleles do not encode functional desaturases, whereas one *SAD2-A* allele has been shown to be functional [24]. However, two further allele groups are unlikely to be functional: one group (*SAD2-D*) had a repetitive sequence insertion at the start of the coding sequence, and one (*SAD6-B*) contained significantly more stop and frame-shift mutations than expected by chance (Table S1).

**Figure 3:**
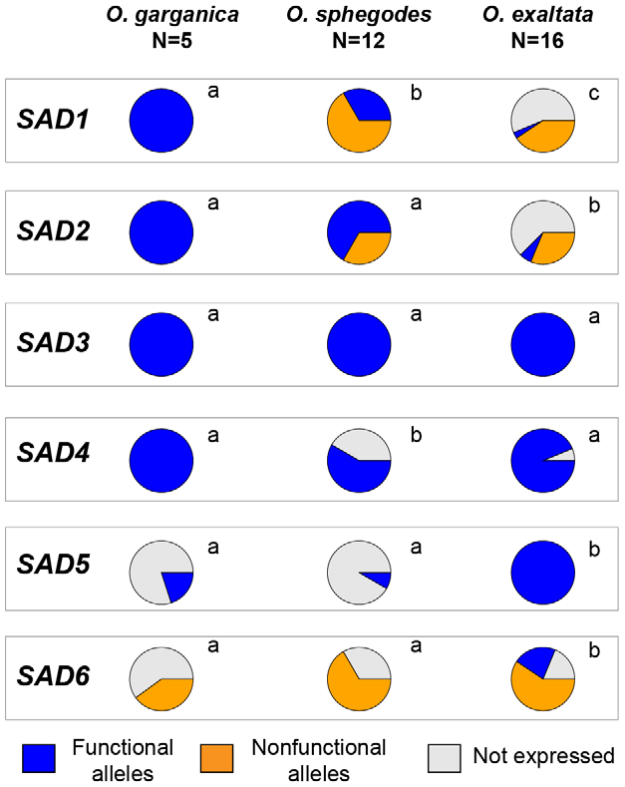
Distribution of *SAD* allele occurrence in three species. Color indicates different allele groups (blue: putatively functional alleles; orange: putatively nonfunctional alleles; gray: alleles not expressed in a given species). Different letters beside pie charts indicate statistical difference (*p*<0.05; χ^2^ test).

Combining putative coding sequence functionality and biochemical activity data, we classified all alleles into three categories: (a) putatively functional and expressed, (b) putatively nonfunctional and expressed, and (c) non-expressed alleles (Figure S3). For *SAD1/2/5/6*, the distributions of these allele categories are significantly different between *O. exaltata* and the other two species (Figure 3). For *SAD1/2*, functional expressed alleles were significantly more common in *O. garganica* and *O. sphegodes* than in *O. exaltata*. By contrast, *SAD5/6* showed the opposite pattern, functional expressed alleles being more common in *O. exaltata*.

To estimate *F*_ST_ values for all expressed *SAD* homologs in all three species, an *in silico* resampling approach was employed (see Methods), treating all individuals as diploids based on flow cytometry data [17]. *O. garganica* was not included in this analysis due to the smaller sample size, and *F*_ST_ could not be calculated for *SAD5* because it was only observed in *O. exaltata*. *SAD2* and *SAD6* showed significantly higher *F*_ST_, (0.44±0.02 and 0.32±0.006 respectively, mean ± standard error, *p*<0.01) than *SAD1*, *SAD3* and *SAD4* (0.22±0.005, 0.25±0.006 and 0.23±0.01 respectively, mean ± standard error).

### Allelic gene expression of *SAD* is associated with alkene composition differences among species

**Figure 4:**
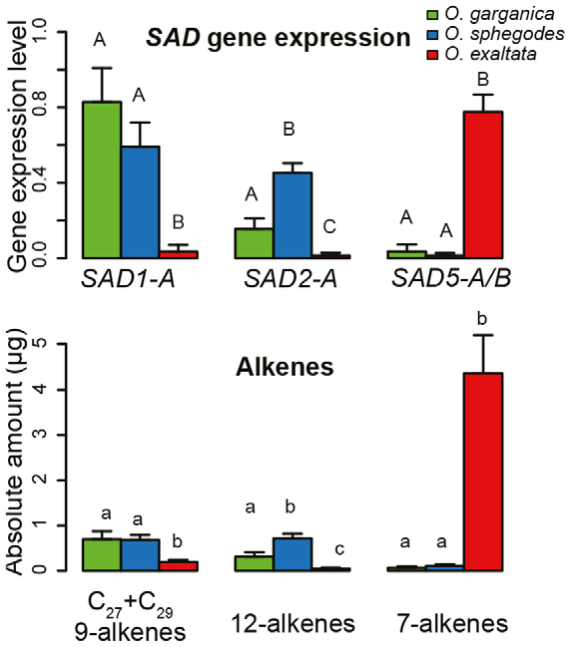
Allelic gene expression and alkene production in different species. *Top*, gene expression of different *SAD* alleles; *Bottom*, alkene levels. Colors indicate species (red, *O. exaltata*; green, *O. garganica*; blue, *O. sphegodes*). Error bars show standard error. Different letters indicate significant differences among species for each allele (*p*<0.05; ANOVA).

Five *SAD* copies (all except *SAD3*) showed divergent species-specific gene expression (Figure S3). Among the alleles of *SAD1*, *SAD1-A* was highly expressed in *O. sphegodes* and *O. garganica* but not in *O. exaltata* (Figure 4), whereas *SAD1-B* was highly expressed in *O. sphegodes* and *O. exaltata* (Figure S3). Among the *SAD2* allele groups, *SAD2-A/B* were highly expressed in both *O. sphegodes* and *O. garganica*, whereas the expression of *SAD2-C/D* was low in all study species (Figure S3). The expression of *SAD4* was high in *O. garganica* and *O. exaltata*, but low in *O. sphegodes*. All alleles of *SAD5* and *SAD6* were highly expressed mostly in *O. exaltata*.

Natural F_1_ hybrids among *O. sphegodes* and *O. exaltata* (identified from AFLP data) showed a similar scent [17] and *SAD* expression pattern to *O. sphegodes* (Figure S3). For *SAD1* and *SAD2*, alleles (*SAD1-A/B*, *SAD2-A/B*) that were most likely inherited from *O. sphegodes* were found to be highly expressed in these F_1_ hybrids, whereas none of the *SAD5/6* alleles was expressed (Figure S3).

Statistical analysis of data from natural populations showed a strong correlation between the expression of specific *SAD* allele groups and alkene production. The expression level of *SAD1-A* was found to be significantly (*p*<0.001) positively correlated with 9-C_29_, 12-C_27_ and 12-C_29_ alkenes; *SAD2-A* was significantly (*p*<0.001) positively correlated with 9-C_27_, 9-C_29_, 12-C_25_, 12-C_27_, and 12-C_29_ alkenes (Figure 2, Figure S4); *SAD5-A/B* were significantly (*p*<0.001) correlated with all 7-alkenes (Figure 2, Figure S4).

### Alkene composition affects pollinator attraction

**Figure 5:**
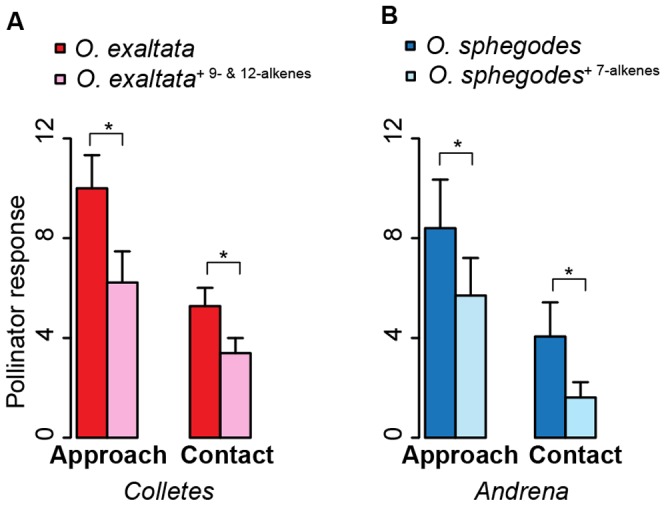
Effects of alkenes with different double-bond position on pollinator behavior. (*A*) Addition of 9- and 12- alkenes (associated with *SAD1/2*) onto *O. exaltata* flowers reduced its pollinator (*C. cunicularius*) response (*N* = 18). Red bar, control *O. exaltata*; pinkish bar, scent manipulated *O. exaltata*. (*B*) Adding 7-alkenes (associated with *SAD5/6*) into *O. sphegodes* floral scent extract reduced its pollinator (*A. nigroaenea*) response (*N* = 17). Blue bar, control floral scent of *O. sphegodes*; light blue bar, manipulated floral scent of *O. sphegodes*. Y-axis refers to number of counts. Asterisks indicate statistical significance (pairwise Mann-Whitney U test): *, *p*<0.05.

To understand the driving force for allelic evolution of *SAD* homologs in *Ophrys*, we tested the effects of alkenes with different double-bond position – associated with different *SAD* homologs – on pollinator behavior. We quantified pollinator responses to control and manipulated flowers and scent extracts of *O. exaltata* and *O. sphegodes*. Addition of 9- and 12-alkenes (associated with *SAD2-A*) to *O. exaltata* labella reduced the attractiveness to its pollinator (the masked bee *Colletes cunicularius*) by about 40% for approach and contact (*N* = 18, *p* = 0.023 and *p* = 0.015 respectively, Wilcoxon signed rank test, Figure 5A). Adding 7-alkenes (associated with *SAD5-A/B*) to floral scent extracts of *O. sphegodes* reduced the attractiveness to its pollinator (the mining bee *Andrena nigroaenea*) by about 30% and 60% for approach and contact respectively (*N* = 17, *p* = 0.028 and *p* = 0.045, Wilcoxon signed rank test, Figure 5B).

## Discussion

### Differences in alkene production are associated with *SAD* gene expression

Our data indicate that alkene biosynthesis is associated with the expression of certain *SAD* homologs in *Ophrys*. SAD2 catalyzes the introduction of a double-bond at position 9 of 18:0-ACP and position 4 of 16:0-ACP [24], producing 18:1ω-9-ACP and 16:1ω-12-ACP, which should eventually lead to the production of 9- and 12-alkenes [23], [24]. The significant correlation of *SAD2* expression with the amounts of certain 9- and 12-alkenes in different plant tissues, floral developmental stages, and natural populations lends further support to this hypothesis. Although biochemical assays suggested that SAD1 (allele group *SAD1-B*) is not catalytically active [24], this may not be true for other *SAD1* alleles (*SAD1-A*) found in both *O. sphegodes* and *O. garganica*, SAD1-A and SAD1-B differing by 14% at the amino acid sequence level. Furthermore, *SAD1-A* expression was significantly correlated with some 9- and 12-alkenes (Figure S4). This indicates that, like *SAD2*, *SAD1* may also contribute to 9- and/or 12-alkene biosynthesis in natural populations.

The significant correlation of *SAD5* expression with the amount of 7-alkenes (Figure 2) suggests that *SAD5* is involved in 7-alkene biosynthesis. In addition, the high sequence identity (>95% at the amino acid level) of *SAD5* to *SAD6-A*, which was highly expressed in one *O. exaltata* population (SPF), indicates that both may have the same enzymatic function. We hypothesize that SAD5/6 introduces a double-bond at position 11 into 18:0-ACP, producing 18:1ω-7-ACP, or at position 9 of 16:0-ACP, producing 16:1ω-7-ACP. Further biochemical studies are required to test this hypothesis. These unsaturated intermediates could then be elongated to produce 7-alkenes. Therefore, changes in the expression of *SAD5/6* could directly lead to different amounts of 7-alkenes in different *Ophrys* species.

### *SAD* homologs are under pollinator-mediated selection

Our data suggest that *SAD* homologs evolve under pollinator-mediated selection, considering that genetic drift is a less likely explanation given large effective population sizes in our study species [11]. The expression of *SAD1/2* was high in both *O. garganica* and *O. sphegodes*, but was very low in most *O. exaltata* individuals (Figure 4, Figure S3). In those very few individuals of *O. exaltata* that did highly express *SAD2*, it either was rendered nonfunctional by a repetitive sequence insertion (*SAD2-D*; population MDL), or had amino acid substitutions located on the surface of the protein (*SAD2-C* allele group), which have been suggested to reduce the activity of SAD2 [24]. This indicates that disruptive selection might act on *SAD2* (in terms of gene expression or overall enzymatic activity) to maintain (in *O. sphegodes* and *O. garganica*) or reduce (in *O. exaltata*) the production of 9- and/or 12-alkenes in *Ophrys* floral odor. Evidence of positive selection on *SAD1/2* detected by *d*_N_/*d*_S_ ratio tests is consistent with this hypothesis (Figure 1 and ref. [24]). Indeed, our pollinator-attraction assay suggests disruptive selection on 9- and/or 12-alkenes among species. This behavioral test demonstrated that adding 9- and 12-alkenes onto the floral labella of *O. exaltata* reduced its attractiveness to the pollinator by about 40% (Figure 5A). This indicates that while 9/12-alkenes act as the main attractants of *O. sphegodes* to its pollinator *A. nigroaenea* [18], these compounds actually reduce the attractiveness of the odor bouquet of *O. exaltata* to its pollinator *C. cunicularius*. Reduced responses of pollinator males to heterospecific odor blends may have evolved under sexual selection for maximum speed and accuracy of finding conspecific females [32]. Therefore, pollinator-imposed disruptive selection acts to change 9/12-alkene composition among these two *Ophrys* species by changing the expression or enzymatic activity of SAD1/2.

However, for *SAD5/6*, the opposite pattern was observed. *SAD5/6* were highly expressed in *O. exaltata*, but hardly expressed in *O. sphegodes* and *O. garganica*. A significantly higher frequency of frame-shifts or premature stop codons was found in *SAD6* of *O. sphegodes* and *O. garganica* (Table S1), indicating that *SAD6* alleles in these two species may be released from purifying selection such that loss-of-function mutations can accumulate. Neither positive selection nor purifying selection was detected on *SAD5/6* using codon-based methods (Figure 1), suggesting that pollinator-mediated selection on 7-alkenes in *O. sphegodes* and *O. garganica* primarily acts on the expression level of *SAD5/6*. Indeed, selection against 7-alkenes in *O. sphegodes* was confirmed by behavioral tests with its pollinator, since addition of 7-alkenes to the floral scent of *O. sphegodes* resulted in a significant reduction in pollinator attraction (Figure 5B). Therefore, while 7-alkenes attract *O. exaltata*'s pollinator [19], [31], they reduce the attractiveness of *O. sphegodes* to its pollinator. Hence, pollinator-mediated disruptive selection may also drive the evolution of 7-alkene quantity in these two *Ophrys* species by changing the expression of *SAD5/6*. In contrast to these genes that are associated with alkene production, *SAD3* and *SAD4*, which were not significantly correlated with alkene occurrence, showed no significant sequence divergence among species (Figure 3, Figure S2), as would be expected for genes that are not targets of selection.

Overall, our data suggest that pollinator adaptation in *Ophrys* is achieved via reciprocal regulation or activity changes of desaturases involved in 7- and 9/12-alkene biosynthesis in response to disruptive selection by different pollinator preferences.

### Both *cis*- and *trans*-regulatory elements may be involved in controlling species-specific alkene compositions

The changes in 7- and 9/12-alkene production that are linked to differences in *SAD* gene expression may be explained by the action of *cis-* or *trans-*acting elements. The expression of *SAD1/2*, which is associated with 9/12-alkene production, differed among *O. exaltata* (weak expression) and *O. sphegodes* (strong expression) (Figure S3). However, two putative F_1_ hybrids only expressed the alleles expected to be inherited from *O. sphegodes*, but not *O. exaltata* (Table S2; Figure S3). This indicates that down-regulation of *SAD1/2* expression in *O. exaltata* might be due to changes in a *cis*-regulatory element (such as a promoter or enhancer). In contrast, although differences in expression of *SAD5/6*, which are associated with 7-alkene production, were found between *O. sphegodes* and *O. exaltata*, the putative F_1_ hybrids did not express either allele expected from the parental species (Table S2; Figure S3). This suppression of expression of *SAD5/6* in F_1_ hybrids indicates that – while additional *cis*-regulatory changes cannot be ruled out – a *trans*-acting factor is likely involved in the different *SAD5/6* gene expression among species. This suggests the presence of a (dominant) suppressor of *SAD5/6* expression in *O. sphegodes* (e.g., a transcriptional repressor or a miRNA reducing *SAD5/6* mRNA levels) that is absent or inactive in *O. exaltata*. However, it is also possible that the dominant expression of *SAD5/6* genes in F_1_ hybrids is due to epigenetic changes upon hybridization [33], [34].

In conclusion, our data based on multiple independent lines of evidence suggest that pollinator adaptation in the three studied *Ophrys* species is largely due to changes in *SAD1/2* and *SAD5/6*, in terms of gene expression and potentially also in terms of the function of their gene products, and that both *cis-* and *trans-*regulation of gene expression contribute to this process. Our data indicate that pollinator adaptation in plants with a specialized pollination system may be due to few changes in the genome, with a large phenotypic effect. Furthermore, because reproductive isolation among closely related *Ophrys* species is mainly a consequence of specific pollinator attraction, such adaptation to different pollinators can directly prevent gene flow and ultimately lead to speciation.

## Materials and Methods

### Plant material

Population samples were collected in southern Italy (Table S3), at the same locations as described in Xu et al. [17], with three additional *O. exaltata* individuals from San Pietro in Fine (SPF) in southern Italy (N41°25′38″, E13°58′04″). Two F_1_ hybrids between *O. exaltata* and *O. sphegodes* were previously identified based on AFLP markers [17]. For each plant individual, one labellum of an unpollinated flower was used for floral odor extraction as described previously [17], and then immediately flash frozen in liquid nitrogen, and stored at −80°C until RNA extraction. For developmental stage- and tissue-specific analysis of hydrocarbons and gene expression, five *O. exaltata* and six *O. sphegodes* individuals grown in a greenhouse at the Botanical Garden of the University of Zürich were used and processed as described previously [24].

### GC and GC/MS analysis

GC and GC/MS analysis, identification and quantification of compounds were performed as described previously [11], with modifications [17]. Discrimination of 11- and 12-alkenes was not possible with the GC parameters used, however, 12-alkenes were earlier determined to be the predominant isomers in *O. sphegodes* [35], [36]. The absolute amounts of alkenes and alkanes with a carbon chain length from 21 to 29 were calculated based on an internal standard. For tissue/stage-specific samples, the relative amount of alkenes was calculated since the use of comparable amounts of tissue could not be ensured.

### RNA extraction, cDNA synthesis, RACE, and RT–PCR

Total RNA was extracted using Trizol reagent (Invitrogen, Carlsbad, USA) following the manufacturer's protocol, and RNA quality and quantity were assessed by agarose gel electrophoresis and spectrophotometry on a NanoDrop ND-1000 (Witec AG, Littau, Switzerland). First strand cDNA was synthesized as described in [24]. To obtain the full-length coding sequence of candidate genes, 5′ RACE was performed as described in [37], with minor modifications [24], and 3′ RACE as in [38]. Gene-specific primers used for RACE are listed in Table S4. Advantage GC 2 DNA Polymerase (Clontech Laboratories Inc, Mountain View, USA) was used for RACE PCR with a touchdown program: 96°C 15 s; 3 cycles of [94°C 20 s, 68°C 3 min 30 s]; 7 cycles of [94°C 20 s, 67°C (1°C decrease per cycle) 30 s, 68°C 3 min 30 s]; 30 cycles of [94°C 25 s, 55°C 30 s, 68°C 3 min 30 s]; final extension at 68°C for 10 min. The amplified fragments were cloned into pDRIVE vector (Qiagen, Hilden, Germany), following the provided protocol. Desaturase homologs were amplified for all cDNA samples, using gene-specific primers containing *att*B adapter sequences (Table S4). RT-PCR was performed in 15 µl reaction volume containing cDNA template equivalent to 15 ng RNA as follows: 95°C 3 min; 33 cycles of [95°C 30 s, 58–60°C 30 s (see Table S4 for annealing temperatures), 72°C 1 min 30 s]; final extension at 72°C for 10 min, using REDTaq ReadyMix (Sigma-Aldrich, St. Louis, USA) mix supplemented with 0.6 units *Pfu* polymerase (Promega AG, Dübendorf, Switzerland). Three µl PCR product were loaded on an agarose gel to confirm amplification.

### Cloning and sequencing

Amplified PCR products from each population of each species were pooled and then purified with Wizard SV Gel and PCR Clean-Up kit (Promega AG, Dübendorf, Switzerland), and recombined into Gateway cloning vector pDONR221 (Invitrogen, Carlsbad, USA) using the manufacturers' protocols. Competent *E. coli* One Shot TOP10 cells (Invitrogen, Carlsbad, USA) were used for transformation. In order to recover all possible alleles, the number of clones picked and screened by PCR was at least three times the number of possible alleles in diploids, for each cloning library. Clones were PCR amplified, purified and sequenced as previously described [24]. All sequences were deposited in GenBank (accession numbers are listed in Table S5).

### Sequence analysis and allele group assignment

Forward and reverse sequences of each clone were assembled and manually edited in SeqMan v7.1.0 (Lasergene DNAstar, Wisconsin, USA). For each *SAD* homolog, the assembled sequences of each clone were aligned using Clustal W [39]. Sequences with less than two nucleotide differences were considered to be the same allele with PCR or sequencing errors, and were merged into one consensus sequence. The consensus sequences and all singleton sequences, which differed by more than two nucleotides, were used for assignment to allele groups. To do so, given the low sequence divergence, a dendrogram was constructed for each *SAD* homolog in MEGA 4.0 [40], using a pairwise distance and the UPGMA method, with pairwise deletion of gaps, and a homogeneous substitution pattern among lineages and sites. Bootstrap analysis was conducted using 1000 pseudo-replicates. Allele groups were assigned based on UPGMA tree topology with bootstrap support (Figure S2). The relationship among alleles was also inferred by Bayesian analysis (Figure S5) in MrBayes (v3.2.1; for details see below) [41], which was congruent and largely confirmed the clusters from UPGMA analysis. The Bayesian analysis showed *SAD2-C* alleles to be nested in *SAD2-B*, and *SAD6-A* in *SAD6-B*, but was in agreement with our primary allele group assignment.

### Measuring gene expression by semi-quantitative PCR

*SAD* gene expression was assessed by semi-quantitative RT-PCR with allele-specific primers (Table S4). PCR was performed in 10 µl reaction volume with cDNA from 12 ng total RNA as a template. Each PCR was performed as: 95°C 3 min; 29 cycles of [95°C 30 s, 58–60°C 30 s (see Table S4 for different primer annealing temperatures), 72°C 1 min 30 s]; final extension at 72°C for 10 min using REDTaq ReadyMix (Sigma-Aldrich, St. Louis, USA). For all RT-PCRs, the putative *Ophrys* housekeeping gene *G3PDH* [24] was used as control. Five µl of each PCR product were loaded on 0.8% agarose gel, recorded and quantified using ImageJ (1.42q) [42] as described in [24].

### Bioassay for pollinator preferences on floral scents

Bioassays for pollinator preferences were performed between beginning of March to middle April 2011 at Capoiale (Southern Italy) and Charrat (Wallis, Switzerland) for *O. exaltata* and *O. sphegodes*, respectively. For both species, pollinator preferences on control and manipulated scent (for *O. exaltata*, 9/12-alkenes added; for *O. sphegodes*, 7-alkenes added) were tested (Table S6 and Table S7). The preference of *O. exaltata*'s pollinator, *C. cunicularius*, was assessed with whole inflorescences (bearing 2–3 flowers) assayed individually. Each inflorescence was placed on bushes where *C. cunicularius* males were abundant; pollinator responses were recorded for 10 minutes. Afterwards, a 9/12-alkene mixture mimicking natural blends occurring in *O. sphegodes* was added onto each floral labellum of the same inflorescence (see Table S6 and Table S7) and the pollinator responses monitored for a further 10 min. For each subsequent test, the plants were placed at a different position to avoid habituation effects often found after multiple subsequent testing at one location. In total, 18 inflorescences that had at least 2 flowers were tested. The preference of the pollinator of *O. sphegodes*, *A. nigroaenea*, was assessed in choice experiments on floral scent with black plastic beads. This different testing procedure was chosen because no natural plants were available at this testing location. The floral scent of floral labella was first extracted with 500 µl hexane [17]. For each choice experiment, 100 µl of floral extract was tested against 100 µl of floral extract of the same flower plus 7-alkene mixture (see Table S6 and Table S7). The dummy was placed on bushes where *A. nigroaenea* males were abundant. Pollinator responses were recorded for six minutes. In total, 17 replicates of this experiment were performed. For all pollinator behavioral tests, pollinator responses were classified into: approach (a zig-zagging or undulating approach towards the tested flowers or beads) and contact (either a short punching contact or landing on the tested flowers or beads) [19].

### Bioinformatics and statistical analysis

All monocot *SAD* sequences were taken from the plant *SAD* homolog data set of [24]. Sequences were re-aligned based on amino acid sequence using Muscle 3.8.31 [43]. Phylogenetic analysis was performed in MrBayes 3.1.2 [41] (burn-in 13 out of 40 million generations) using the GTR+I+G model, which was estimated to be the best model by MrModeltest (2.3; AIC criterion) [44]. All sequence data were partitioned by codon positions, and MrBayes analysis performed using one cold and three heated chains, trees sampled every 1000 generations, and combined into a 50% majority rule consensus tree. The signature of selection on selected branches was tested using PAML 4.4 [45], as previously described [24]. The significance of different amounts of floral odor and gene expression among species was assessed by ANOVA after normality testing of the data distribution by the Shapiro test [46]. Differences in allele distribution among different species were assessed by χ^2^-testing. To estimate the pattern of divergence for each *SAD* homolog, we first genotyped each *Ophrys* individual by allele-specific RT-PCR; then we randomly sampled two sequences from the allele groups based on species and population information. This procedure was repeated 100 times for *in silico* re-sampling. *F*_ST_ was calculated using Arlsumstat, a modified version of Arlequin (v.3.5.1.3) [47]. The association between floral scent and gene expression in natural populations was assessed using a generalized linear model (GLM) and a linear mixed-effect model (LME) with population as random factor. These models were simplified by stepwise removal of factors using the stepAIC method [48]. For the tissue/stage-specific dataset, the relative amount of each floral scent compound was used (arcsine square-root transformed) as described by Schlüter et al. [24], since the size of floral labella varies in different developmental stages. For the population data set, the absolute amount of each floral scent compound was used. The significance of the presence of nonfunctional alleles in different allele groups was tested using Fisher's exact test. All statistical analyses were performed in R 2.11.0 [49].

## Supporting Information

Figure S1 Gene expression and floral odor in different plant tissues. (*A*, *B* and *C*), relative amount (as proportion of hydrocarbons) of different alkenes in floral labella (*A*), sepals & petals (*B*), and leaf (C) tissue of *O. sphegodes* or *O. exaltata*; (*D*, *E* and *F*), normalized gene expression of the five *SAD* homologs in floral labella (*SAD6* was not expressed in these individuals) (*D*), sepals & petals (*E*), and leaf (*F*) tissue of *O. sphegodes* and *O. exaltata*. Error bars indicate the standard error. Asterisks indicate significant differences between species (*p*<0.05, one-way ANOVA).
(TIF)

Figure S2 Dendrograms of *SAD1*, *SAD2*, *SAD3*, *SAD4*, and *SAD5/6* produced using the UPGMA method in MEGA (v. 4.0). Symbol color refers to the species from which sequences were obtained, while symbol shape indicates the source population. Blue, *O. sphegodes*; red, *O. exaltata*; green, *O. garganica*; pink, F_1_ hybrids of *O. sphegodes* and *O. exaltata*; black, consensus sequences from at least two species. Numbers on branches are bootstrap values. An asterisk (*) indicates stop codon or frame-shift mutations in the sequence. Sequences included for PAML analysis shown in Figure 1 are marked with “#".
(TIF)

Figure S3 Allelic gene expression of six *SAD* homologs in natural populations of the study species. The height of each bar indicates mean normalized expression of each allele, and error bars indicate standard error. Letters on each bar indicate statistical significance comparing among species within each allele group (*p*<0.05, one-way ANOVA).
(TIF)

Figure S4 Statistical summary of associations among gene expression of *SAD* homologs and each alkene. GLM and LME indicate different statistical methods, Generalized Linear Model and Linear Mixed-Effects model, respectively. (*A*) Relative expression of *SAD1*–*SAD5* versus relative amount of each alkene among different floral tissues/stages. Relative amount of alkenes was used after *f(x) = arcsin x^0.5^* transformation. (*B*) Allelic expression of *SAD1*–*SAD6* versus absolute amount of each alkene among species/populations. Absolute amount of alkenes (in µg) was used after *f(x) = ln (x+0.01)* transformation in the population dataset.
(TIF)

Figure S5 Phylogenetic tree of *SAD1*, *SAD2*, *SAD3*, *SAD4*, and *SAD5/6* using Bayesian inference in MrBayes (v3.2.1). Sequence data were partitioned by codon positions. The analysis used one cold and three heated chains, trees were sampled every 1000 generations, and combined into a 50% majority rule consensus tree, discarding trees from the ‘burn-in’ period. Symbol color refers to the species from which sequences were obtained, while symbol shape indicates the source population. Blue, *O. sphegodes*; red, *O. exaltata*; green, *O. garganica*; pink, F_1_ hybrids of *O. sphegodes* and *O. exaltata*; black, consensus sequences from at least two species. Numbers at branches are posterior probability values. An asterisk (*) indicates stop codon or frame-shift mutations in the sequence. Sequences included for PAML analysis shown in Figure 1 are marked with “#".
(TIF)

Table S1 Number of sequences in a given allele group in which frame-shift or stop codon mutations were found. All sequences were classified into two groups: putatively functional and nonfunctional, based on whether frame-shifts/stop codons were observed. Asterisks indicate the proportion of nonfunctional sequences where significantly higher than expected, using Fisher's exact test [*p* cut-off value 0.05].
(XLS)

Table S2 Allelic gene expression in different species. The number in each cell refers to the number of individuals that showed expression of a certain allele group. N refers to the total number of individuals assessed. E, G and S refer to *O. exaltata*, *O. garganica* and *O. sphegodes*, respectively. E×S refers to F_1_ hybrids among *O. sphegodes* and *O. exaltata* (identified by Xu et al. [17], according to molecular maker data; direction of the cross unknown).
(XLS)

Table S3 Plant samples collected in this study. Numbers in cells refer to the number of individuals. Hybrid refers to F_1_ hybrids between *O. sphegodes* and *O. exaltata* as assigned based on AFLP data in previous study [17]. Location information for each population is given in the main text.
(XLS)

Table S4 Oligonucleotides used in this study. For primers compatible with Gateway (Invitrogen) cloning, the full *att*B sites were introduced as described in Invitrogen's manuals. T_A_ refers to the annealing temperature used for PCR reactions.
(XLS)

Table S5 Accession numbers of all sequences obtained in this study.
(XLS)

Table S6 Alkene compositions of *O. exaltata*, *O. sphegodes*, and alkene mixtures used for pollinator tests. For *O. sphegodes* and *O. exaltata*, mean ± SE absolute amounts in individual compounds per 100 µl odor extract (in ng) are shown. For alkenes mixtures that were used for odor manipulation per 100 µl odor extract, absolute amounts (in ng) of the compounds are shown.
(XLS)

Table S7 Relative amounts (%) of 7-alkenes and 9/12-alkenes in control and manipulated floral odor bouquets used for pollinator behavior tests. Mean ± standard error for each compounds are listed.
(XLS)
