# Supplementary figures and images for: The Genetic Basis of Pollinator Adaptation in a Sexually Deceptive Orchid
Source: PLoS Genet. 2012 Aug 16;8(8):e1002889. doi: 10.1371/journal.pgen.1002889 (PMC3420943; doi:10.1371/journal.pgen.1002889)

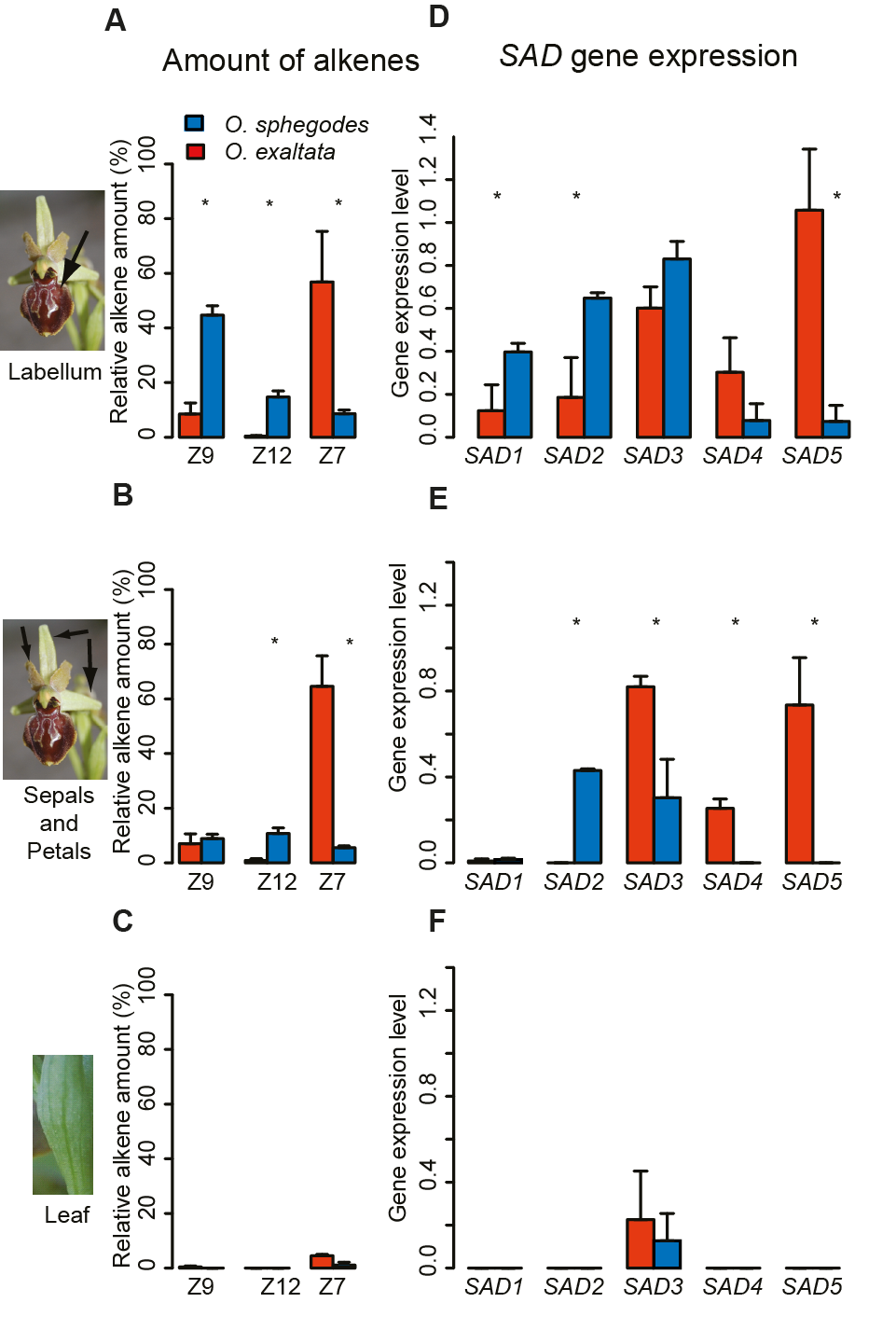

Supplement: Figure S1 — Gene expression and floral odor in different plant tissues. (A, B and C), relative amount (as proportion of hydrocarbons) of different alkenes in floral labella (A), sepals & petals (B), and leaf (C) tissue of O. sphegodes or O. exaltata; (D, E and F), normalized gene expression of the five SAD homologs in floral labella (SAD6 was not expressed in these individuals) (D), sepals & petals (E), and leaf (F) tissue of O. sphegodes and O. exaltata. Error bars indicate the standard error. Asterisks indicate significant differences between species (p<0.05, one-way ANOVA). (TIF) [file pgen.1002889.s001.tif]

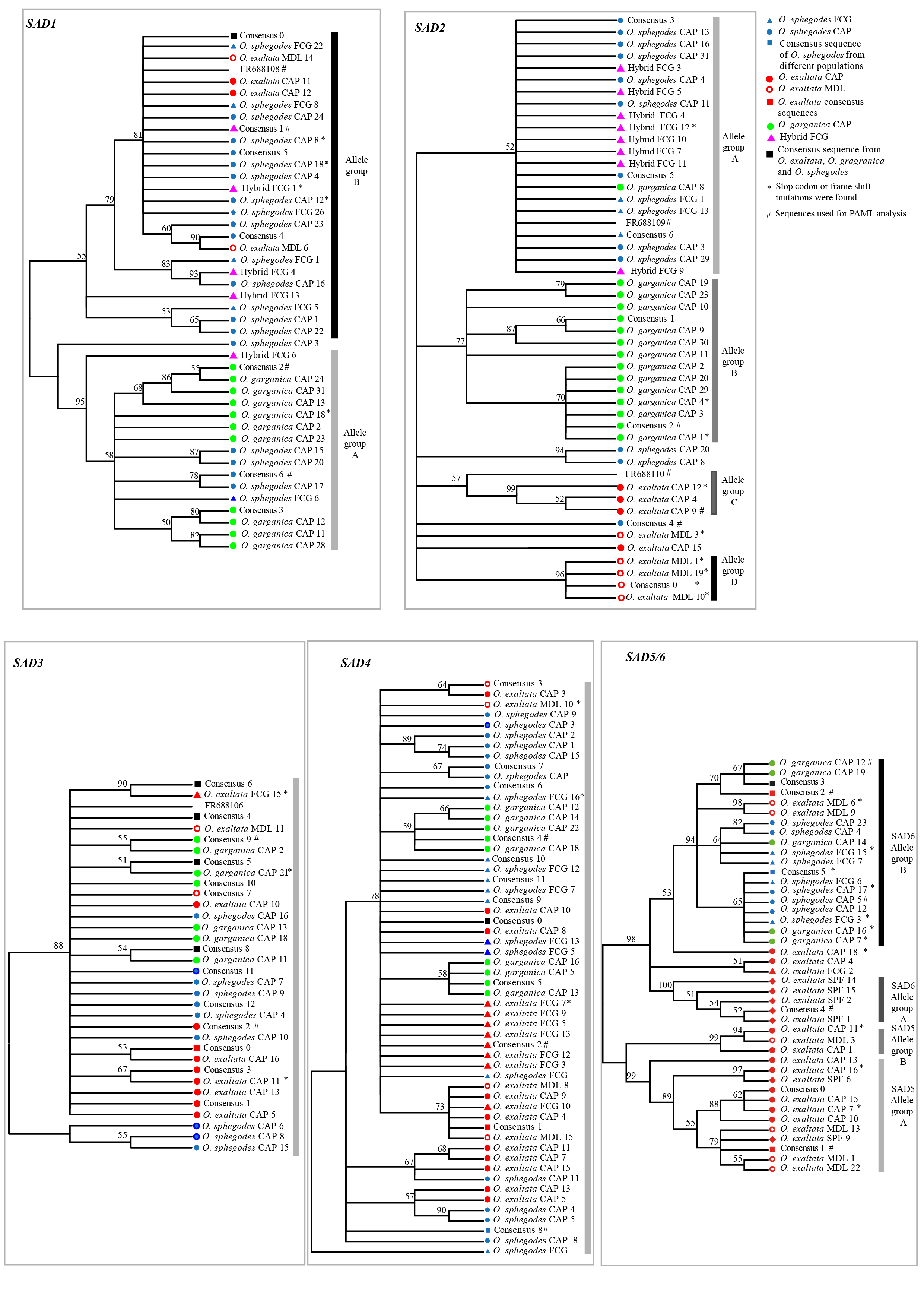

Supplement: Figure S2 — Dendrograms of SAD1, SAD2, SAD3, SAD4, and SAD5/6 produced using the UPGMA method in MEGA (v. 4.0). Symbol color refers to the species from which sequences were obtained, while symbol shape indicates the source population. Blue, O. sphegodes; red, O. exaltata; green, O. garganica; pink, F1 hybrids of O. sphegodes and O. exaltata; black, consensus sequences from at least two species. Numbers on branches are bootstrap values. An asterisk (*) indicates stop codon or frame-shift mutations in the sequence. Sequences included for PAML analysis shown in Figure 1 are marked with “#". (TIF) [file pgen.1002889.s002.tif]

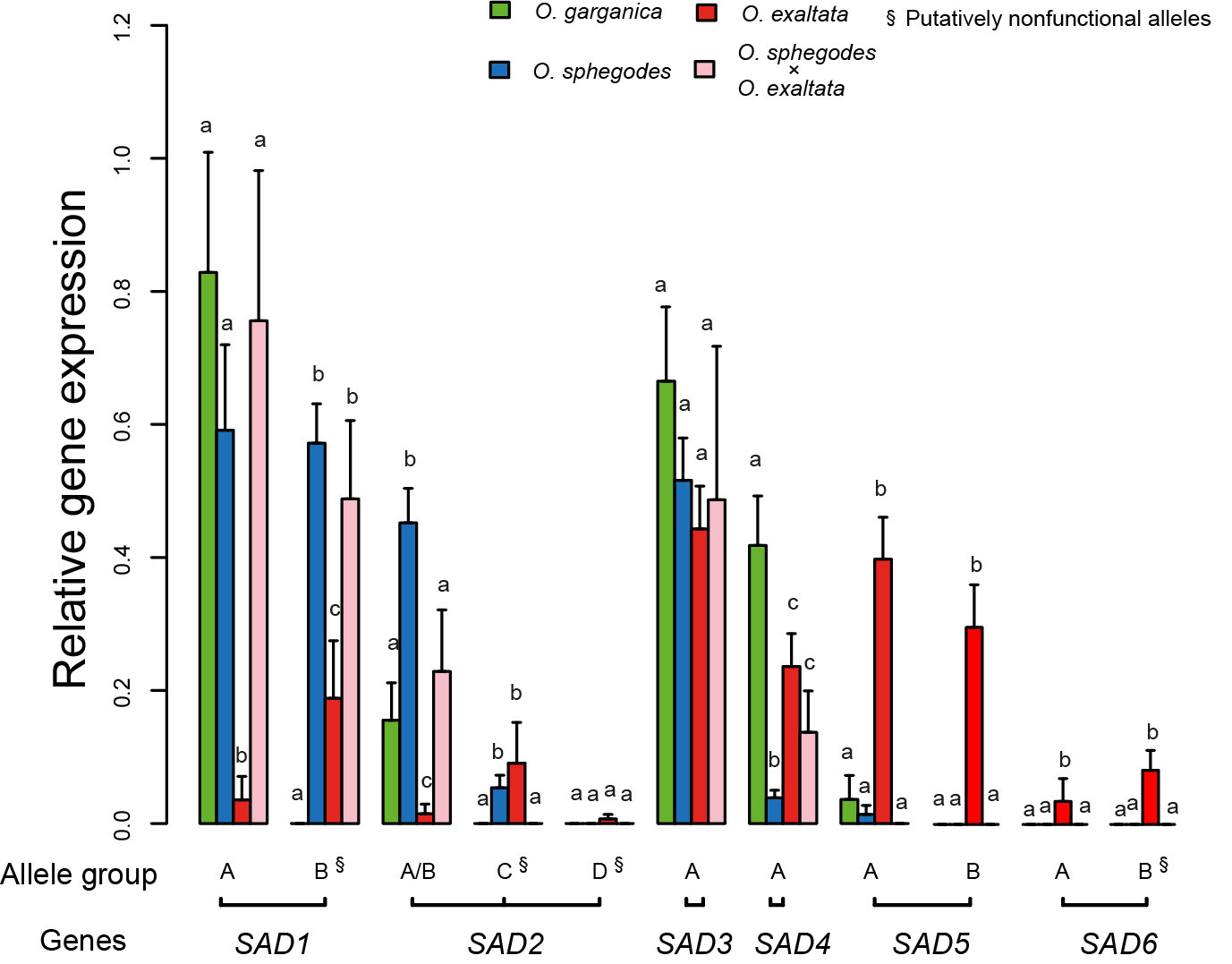

Supplement: Figure S3 — Allelic gene expression of six SAD homologs in natural populations of the study species. The height of each bar indicates mean normalized expression of each allele, and error bars indicate standard error. Letters on each bar indicate statistical significance comparing among species within each allele group (p<0.05, one-way ANOVA). (TIF) [file pgen.1002889.s003.tif]

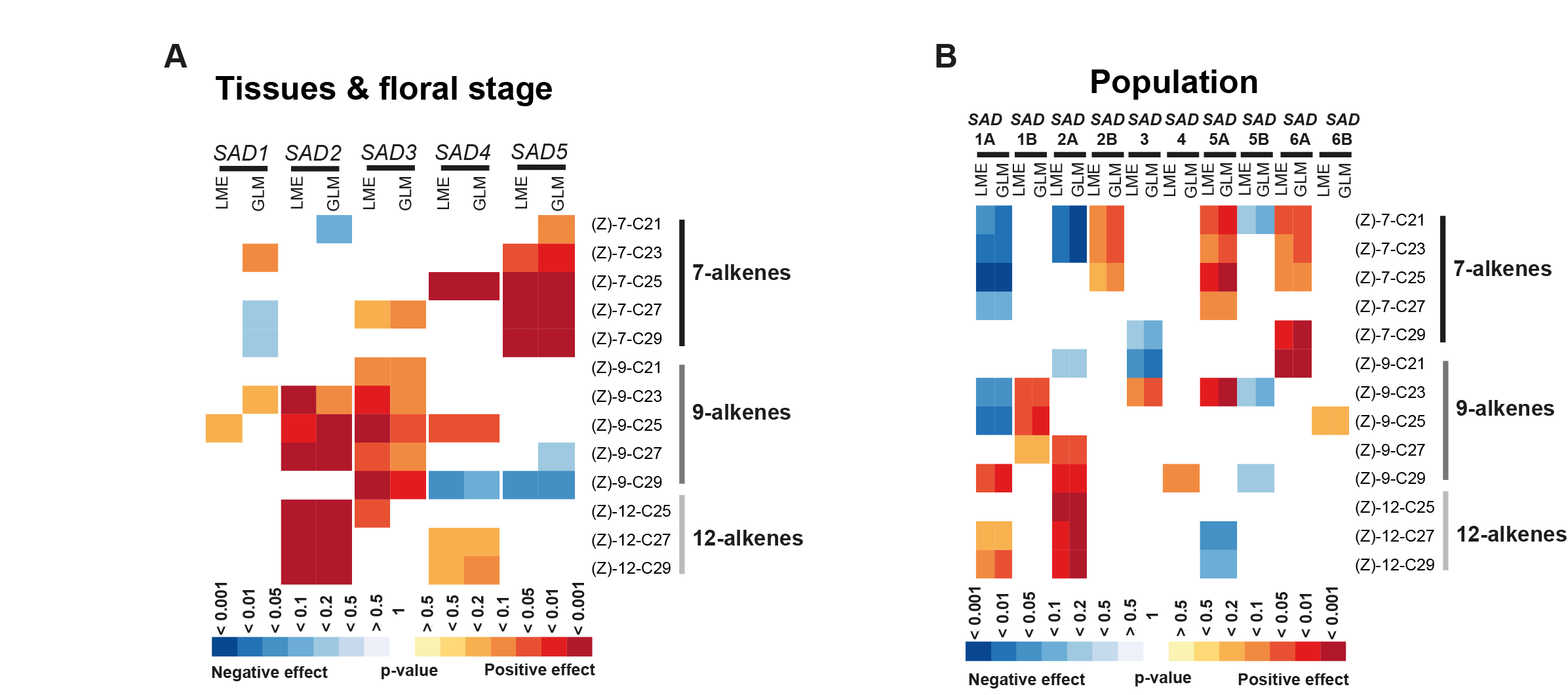

Supplement: Figure S4 — Statistical summary of associations among gene expression of SAD homologs and each alkene. GLM and LME indicate different statistical methods, Generalized Linear Model and Linear Mixed-Effects model, respectively. (A) Relative expression of SAD1–SAD5 versus relative amount of each alkene among different floral tissues/stages. Relative amount of alkenes was used after f(x) = arcsin x0.5 transformation. (B) Allelic expression of SAD1–SAD6 versus absolute amount of each alkene among species/populations. Absolute amount of alkenes (in µg) was used after f(x) = ln (x+0.01) transformation in the population dataset. (TIF) [file pgen.1002889.s004.tif]

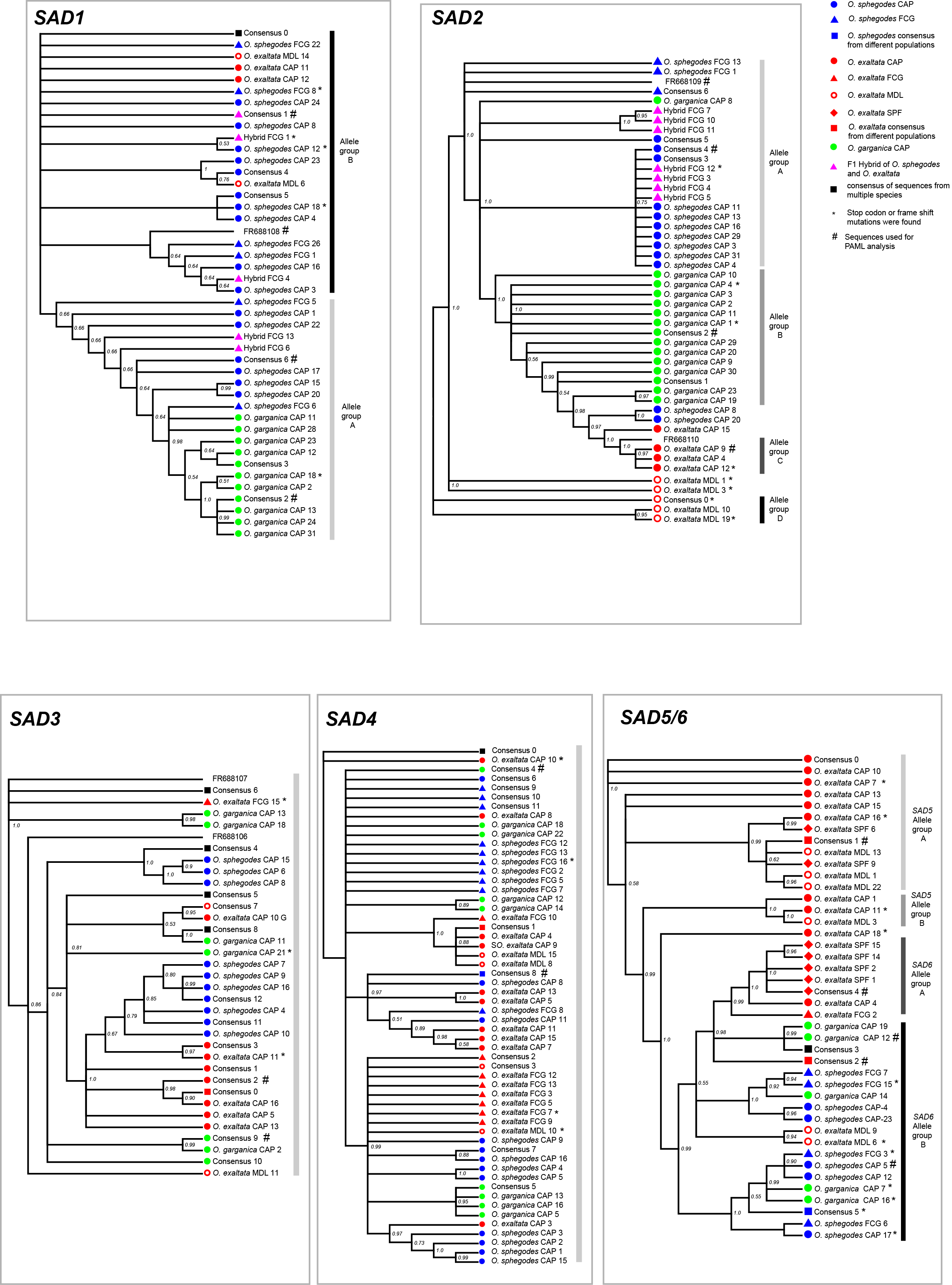

Supplement: Figure S5 — Phylogenetic tree of SAD1, SAD2, SAD3, SAD4, and SAD5/6 using Bayesian inference in MrBayes (v3.2.1). Sequence data were partitioned by codon positions. The analysis used one cold and three heated chains, trees were sampled every 1000 generations, and combined into a 50% majority rule consensus tree, discarding trees from the ‘burn-in’ period. Symbol color refers to the species from which sequences were obtained, while symbol shape indicates the source population. Blue, O. sphegodes; red, O. exaltata; green, O. garganica; pink, F1 hybrids of O. sphegodes and O. exaltata; black, consensus sequences from at least two species. Numbers at branches are posterior probability values. An asterisk (*) indicates stop codon or frame-shift mutations in the sequence. Sequences included for PAML analysis shown in Figure 1 are marked with “#". (TIF) [file pgen.1002889.s005.tif]
